# Time of Initiating Enzyme Replacement Therapy Affects Immune Abnormalities and Disease Severity in Patients with Gaucher Disease

**DOI:** 10.1371/journal.pone.0168135

**Published:** 2016-12-12

**Authors:** Renuka Pudi Limgala, Chidima Ioanou, Matthew Plassmeyer, Mark Ryherd, Lina Kozhaya, Lauren Austin, Cem Abidoglu, Derya Unutmaz, Oral Alpan, Ozlem Goker-Alpan

**Affiliations:** 1 Lysosomal and Rare Disorders Research and Treatment Center, Fairfax, Virginia, United States of America; 2 Amerimmune, O and O Alpan, LLC, Fairfax, Virginia, United States of America; 3 Jackson Laboratory for Genomic Medicine, Farmington, Connecticut, United States of America; Azienda Ospedaliero-Universitaria Santa Maria della Misericordia, ITALY

## Abstract

Gaucher disease (GD) patients often present with abnormalities in immune response that may be the result of alterations in cellular and/or humoral immunity. However, how the treatment and clinical features of patients impact the perturbation of their immunological status remains unclear. To address this, we assessed the immune profile of 26 GD patients who were part of an enzyme replacement therapy (ERT) study. Patients were evaluated clinically for onset of GD symptoms, duration of therapy and validated outcome measures for ERT. According to DS3 disease severity scoring system criteria, they were assigned to have mild, moderate or severe GD. Flow cytometry based immunophenotyping was performed to analyze subsets of T, B, NK, NKT and dendritic cells. GD patients showed multiple types of immune abnormalities associated to T and B lymphocytes with respect to their subpopulations as well as memory and activation markers. Skewing of CD4 and CD8 T cell numbers resulting in lower CD4/CD8 ratio and an increase in overall T cell activation were observed. A decrease in the overall B cells and an increase in NK and NKT cells were noted in the GD patients compared to controls. These immune alterations do not correlate with GD clinical type or level of biomarkers. However, subjects with persistent immune alterations, especially in B cells and DCs correlate with longer delay in initiation of ERT (ΔTX). Thus, while ERT may reverse some of these immune abnormalities, the immune cell alterations become persistent if therapy is further delayed. These findings have important implications in understanding the immune disruptions before and after treatment of GD patients.

## Introduction

Gaucher disease (GD) is caused by a genetic deficiency of the lysosomal enzyme, glucocerebrosidase leading to accumulation of glycosphingolipids in various organ systems, most notably in cells of mononuclear phagocyte system. As a result, most of the immune studies in GD patients have been focused on monocyte/macrophage lineage [1, 2]. However considering that clinical manifestations of GD affect various organ systems, it is important to understand possible dysregulations in major immune cell subsets, such as T-/B- lymphocytes, natural killer (NK) cells and dendritic cells. Moreover, most of the studies relating immune dysfunctions in GD have been performed on animal models. Studies on B-cell abnormalities have been limited to predisposition for monoclonal gammopathies and multiple myeloma in GD [3, 4]. Secretion of several chemotactic factors and corresponding immunological cell invasion has been demonstrated in murine model [5]. Major disease effectors are believed to be cells of macrophage lineage because of their secretion of numerous cytokines and chemokines that influence other poorly defined immunological cell populations. Increases in several such populations were identified in a Gba1 mouse model of GD including antigen presenting cells (APCs), i.e., macrophages, dendritic cells, neutrophils, and T helper cells. Elevated activation of T cells and APCs has also been shown [6]. Even though animal models recapitulating GD have been a source for investigating underlying cellular mechanisms; it is not clear how these findings translate to patients with GD.

Macrophage directed Enzyme replacement therapy (ERT) has been the most accepted form of treatment for GD [7–9]. Therapeutic goals for patients with GD on ERT have been well established, and involve changes in liver and spleen size, improvement in hematological parameters, bone pain and bone crises [10, 11]. However, less than 50% of patients with GD on therapy are expected to meet all these therapeutic goals [12]. Similar to the outcome measures, for monitoring GD patients, a validated disease severity scoring system (DS3) has been defined earlier [13, 14]. This system included data from bone, hematologic and visceral domains, individual items from routine assessments and bone evaluations.

In the present study we determined the immune alterations that persist in GD patients despite ERT and how they relate to individual DS3 scores. We also assessed the role of delay in initiation of therapy (ΔTX) in GD patients, which can correlate with symptoms like avascular necrosis and other complications [15, 16], versus immune alterations.

## Materials and Methods

### Subjects

The handling of tissue samples and patient data was approved by the internal review board (Copernicus Group Independent Review Board) (NCT01358188) including the procedure whereby all patients gave informed consent to participate in this study. Written informed consent was obtained using IRB approved informed consent form (ICF). This procedure was documented via an informed consent progress note which is stored with the original ICF and any other applicable source documents. Of the 31 enrolled subjects with confirmed GD, the immunologic effects of enzyme replacement therapy (ERT) on immunity were assessed in 26 patients (19F/8M, mean age 41yr). Patients were assessed clinically for onset of GD symptoms, duration of therapy, as well as other validated outcome measures for ERT including hematological parameters, presence of liver and spleen enlargement, bone involvement, and for other comorbidities as previously described [10] and then were assigned at their current state to have mild, moderate and severe GD according to DS3 disease severity scoring system [13].

### Immunophenotyping

Direct immunofluorescence with specific antibodies was performed either on peripheral blood or from isolated peripheral blood mononuclear cells (PBMCs) as previously described [17–19]. For staining from whole blood, 100ul of washed blood was used per tube, and samples were acquired on Accuri C6 flow cytometer (BD Bioscience, San Jose, CA) and analyzed using FCS express software (De Novo software, Glendale, CA). For staining using PBMCs, 400,000 cells were used per tube and acquired on LSR II (BD Bioscience, San Jose, CA), while analysis was performed using FlowJo analysis software (FlowJo LLC, Ashland, OR). Detailed gating strategies and identification of major subpopulations is described in supporting data (S1–S5 Figs).

### Statistical analysis

All statistical analysis was performed using GraphPad Prism software (GraphPad Software, Inc., La Jolla, CA). Individual fractions of each cell type from GD patients were compared to normal values calculated from non-GD age and gender matched controls (n = 9) and graphs were generated as Box & Whisker (Tukey) plots. Scatter plots were used for correlation graphs. Pearson correlation was performed to analyze linear relation between ΔTX and DS3 scores. P-values were calculated using two-tailed unpaired Student’s t-test. *: P<0.05; **: P<0.01; ***: P<0.001.

## Results

### Clinical study/scoring

**Fig 1:**
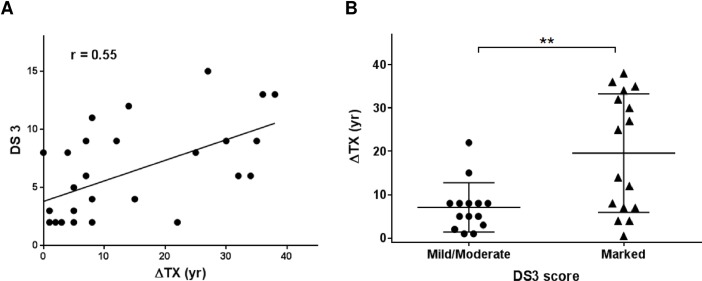
Correlation between delay in treatment initiation and disease severity scores. Delay in initiation of treatment for GD (ΔTX) was plotted against disease severity score (DS3) for each patient. The trend line shows a positive Pearson correlation coefficient between the two values (r value = 0.55, P = 0.0018) (A). Subjects were divided based on their DS3 scores as being mild/moderate (DS3<5) or marked (DS3>6) and their ΔTX values were plotted. Unpaired Student’s t-test revealed P value = 0.0036 (**) indicating very significant difference (B).

Thirty one patients with confirmed GD were enrolled in an IRB approved study (8M: 23F). Demographics and clinical characteristics are summarized in Table 1. Ages at which the symptoms started manifesting (SX), and age at which the treatment for GD was initiated (TX) are noted. There is a lack of accurate assessment of disease burden at the onset of symptoms, however considering that the subjects start therapy when the symptoms become severe, delay in ERT (ΔTX) calculated as the years between TX and SX was taken as one of the parameters of disease burden. For subject ids 13, 29 and 31, who were not under any therapy for GD at the time of study, ΔTX was calculated as the years between current age and SX. Each patient was clinically evaluated for various disease domains including bone disease, hematologic parameters and visceral involvement including organomegaly and GD related pulmonary disease. A disease severity score (DS3) was ascertained for each patient according to previously established guidelines[13] and a composite score was obtained by adding ΔTX and DS3 (Tables 1&2). To assess the effect of the time of initiation of treatment for GD and the disease severity, the delay in ERT (ΔTX) was plotted against DS3 for each patient. Pearson correlation coefficient shows a positive correlation between the two values with r value of 0.55 (P = 0.0018) indicating that greater the delay in therapy, more severe are the disease symptoms (Fig 1A). Subjects were divided based on their DS3 scores to be mild to moderate (<5) and marked (>6) and plotted against their corresponding ΔTX in years. Those with marked DS3 scores had higher ΔTX than those with mild to moderate DS3 scores (P = 0.0036) clearly showing that delay in initiation of therapy resulted in persistently higher manifestation of symptoms (Fig 1B).

**Table 1:** Demographic and clinical data from 31 GD patients enrolled into IRB approved study.

Subject no.	Sex	Genotype	Age (yr)	Age at Symptom onset (SX)	Age at Treatment initiation (TX)	Years in Treatment	ΔTX = TX-SX	DS3 score	Composite score (ΔTX+DS3)
01†	F	N370S/N370S	41	15	18	23	3	2	5
02†	M	N370S/N370S	64	30	42	22	12	9	21
03	F	N370S/L444P	61	5	41	20	36	13	49
04	M	N370S/N370S	47	5	40	7	35	9	44
05	F	RecNci1/N370S	46	2	40	6	38	13	51
06†	F	N370S/N370S	45	18	40	5	22	2	24
07	F	R463C/RecNci1	63	13	43	20	30	9	39
08	F	N370S/N370S	64	33	38	26	5	5	10
09	F	N370S/N370S	57	26	31	26	5	3	8
10	M	N370S/N370S	60	39	54	6	15	4	19
11	M	N370S/N370S	68	18	50	18	32	6	38
12	F	RecNci1/R463C+Rec7	39	4	12	27	8	11	19
13	F	N370S/R496H	58	50	N/A	N/A	8*	4	12
14	F	N370S/c.84insG	61	7	34	27	27	15	42
15†	F	N370S/N370S	25	4	5	20	1	3	4
16	F	N370S/L444P	35	7	11	24	4	8	12
17†	F	N370S/C23Y	42	11	19	23	8	2	10
18	M	N188S/S107L	19	1	8	11	7	9	16
19	F	L444P/L444P	21	1	15	6	14	12	26
20	F	N370S/R120Q	35	2	6	29	4	8	12
21	F	L444P/N370S	27	10	12	14	2	2	4
22	F	L444P/N370S	24	8	9	14	1	2	3
23	M	L444P/N370S	16	3	11	4	8	2	10
24	M	N370S/N370S	50	6	40	10	34	6	40
25	M	N370S/N370S	13	N/A	N/A	N/A	N/A	1	1
26	F	L444P/R463C	52	5	30	22	25	8	33
27	F	N370S/R463C	35	3	11	24	8	2	10
28	F	N370S/R463C	37	5	13	24	8	2	10
29	F	N370S/R496H	9	4	N/A	N/A	5*	2	7
30	F	L444P/L444P	20	0.5	0.5	19.5	0	8	8
31	F	N370S/N370S	28	19	19	2**	7*	6	13

Listed are the sex, causative genotype, age at onset of symptoms (SX), age at the start of treatment (TX). Delay in initiation of therapy (ΔTX) for GD was calculated as difference in years between the age at which the treatment was initiated and age of onset of symptoms (TX-SX). N/A: not applicable.

†: Not included in immunophenotyping analysis.

*: Subjects who were not under any treatment for GD and hence ΔTX was calculated as difference in their age at the time of study and SX.

**: Subject 31 discontinued the treatment after two years of therapy at the age of 21 yr. In this case, ΔTX was calculated as the number of years the subject remained untreated. Disease severity score was ascertained for each patient (DS3) and a composite score was obtained by adding ΔTX and DS3.

**Table 2:** Clinical evaluations and DS3 scoring.

Subject no.	Bone disease	Hematologic parameters	Visceral involvement		DS3 score
			Organomegaly	Pulmonary	
01†	0	+	+	0	2
02†	+++	++	Splenectomy	0	9
03	+++	+	Splenectomy	+	13
04	+++	+	Splenectomy	0	9
05	+/++	++/+++	Splenectomy	0	13
06†	+	+	Splenectomy	0	2
07	+++	+	Splenectomy	0	9
08	+	+	+	0	5
09	+	0	+	0	3
10	+	0	+	0	4
11	++	+	+	0	6
12	+++	+	Splenectomy	+	11
13	+	+	0	0	4
14	+++	+/++	Splenectomy	0	15
15†	+	+	+	0	3
16	+	++	++	0	8
17†	0	+	+	0	2
18	++	+	+/++	+	9
19	+/++	+	+	+	12
20	++	+	+	0	8
21	+	0	+	0	2
22	+	0	+	0	2
23	+	0	+	0	2
24	++	+	++	0	6
25	+	0	+	0	1
26	++	+	Splenectomy	0	8
27	+	0	+	0	2
28	+	0	+	0	2
29	+	0	+	0	2
30	+/++	+	+	+	8
31	++	++	++	0	6

Clinical domains including bone disease, hematologic parameters and visceral involvement (adapted from Gaucher disease severity scoring instrument (DS3) were scored for each patient. A relative disease activity score was assigned as mild (+), moderate (++) and severe (+++) according to the numerical composite score for each domain. Note that (+) denotes the presence of pulmonary involvement.

### Immune abnormalities persist in patients with GD even after long term therapy

Flow cytometry based immunophenotyping was performed on peripheral blood cells from 26 GD patients as well as non-GD controls (n = 9). The absolute numbers were found within the normal reference ranges. However, statistically significant alterations in percentages of specific immune subsets were observed and hence analyzed further. The results with relevant immune subsets are summarized in S1 Table. Lymphocytes were identified from whole blood by gating on CD45 + cells. Lymphocytes comprise of T-, B- and NK cells which were classified by positive expression of CD3, CD20 and CD16/CD56 respectively and as such their percentages together should add up to 100%. Dendritic cells were defined as lineage 1-negative (CD3, CD14, CD16, CD19, CD20 and CD56), CD34-negative, and HLA-DR positive and expressed as fraction of total leukocytes (WBCs).

**Fig 2:**
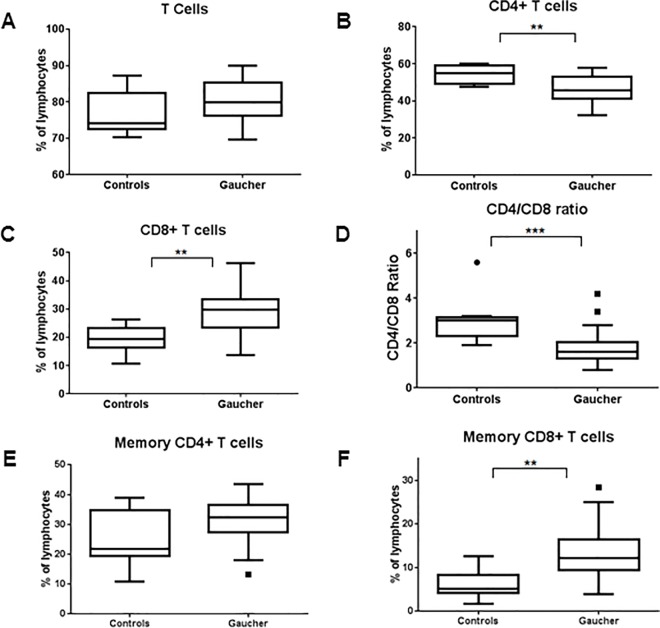
T-lymphocytes and subsets in GD patients. T-lymphocytes (CD3+) from peripheral blood of GD patients and normal controls were assessed using flow cytometry and plotted as fraction of total lymphocytes (A). T helper cells (CD3+/CD4+) (B) and cytotoxic T cells (CD3+/CD8+) (C) were calculated and a ratio of CD4 to CD8 cells was plotted (D). Similarly memory subsets of CD4 (CD3+/CD4+/CD45RO+) and CD8 T cells (CD3+/CD8+/CD45RO+) were calculated and plotted against normal controls (E, F). Unpaired student’s t-test was performed to calculate significance values and included in the plots where significant difference between GD and normal controls was observed.

Overall percentage of T cells did not differ between GD and normal controls (Fig 2A). However significant differences were observed when T cells were fractionated into CD4+T helper cells (Th cells) and CD8+ cytotoxic T cells (Tc cells). A significant decrease in CD4+ cells were observed in GD patients (P = 0.0075), while there was a corresponding increase in CD8+ cells (P = 0.0011) (Fig 2B&2C). CD4/CD8 ratio was then calculated as ratio of CD4+ T helper cells to CD8+ cytotoxic T cells. Lower CD4/CD8 ratio can suggest chronic inflammation or perturbation in the immune system and is observed in conditions like viral infections and immune reconstitution inflammatory syndrome [20–22]. Interestingly, GD patients showed significantly lower (P = 0.0008) CD4/CD8 ratio compared to normal controls (Fig 2D). Memory CD4 and CD8 T cells were assayed by presence of memory cell marker CD45RO. While no change in memory CD4 T cells was observed, GD patients showed much higher number of memory CD8 T cells (P = 0.0028) (Fig 2E&2F).

**Fig 3:**
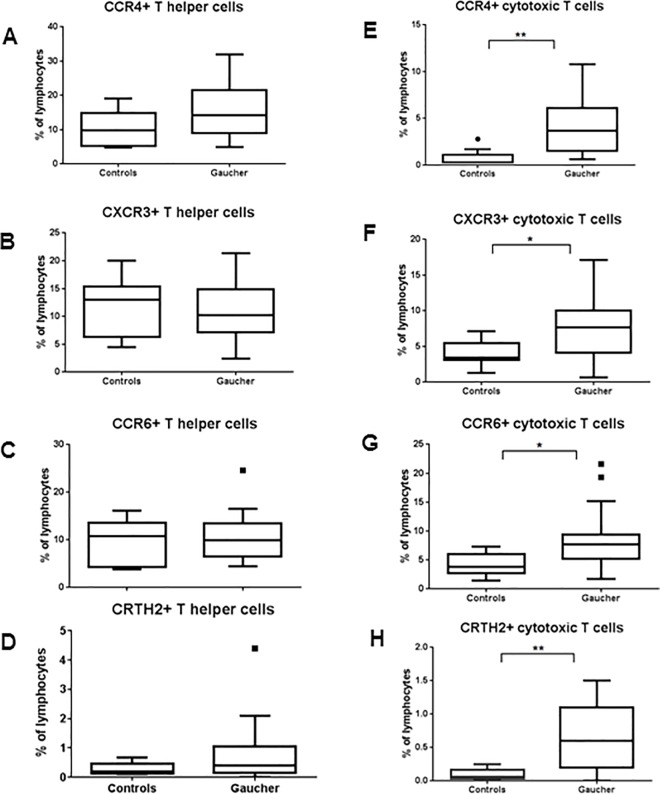
Activation markers in Th and Tc cells. CD4+ T (A) and CD8+ T cells (B) were further analyzed for expression of activation markers (CCR4, CXCR3), chemokine receptor (CCR6) and chemoattractant (CRTH2) which play a role in T-cell mediated inflammation.

**Fig 4:**
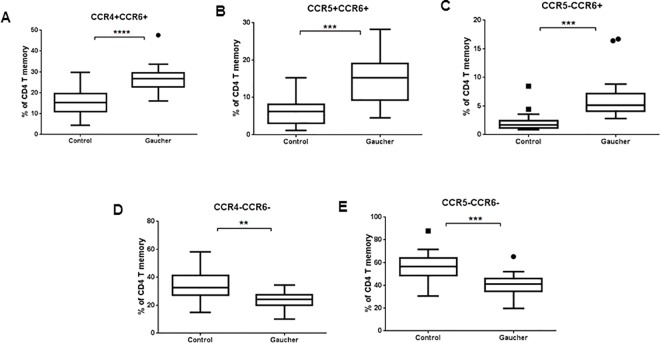
Inflammatory cytokine expression on T cells. Memory T cells were further analyzed for co-expression of inflammatory chemokine receptors (CCR4, CCR5 and CCR6) which play a role in T cell mediated inflammation.

CD4 and CD8 T cells were further analyzed for chemokine receptors CCR4 and CXCR3 and CCR6, and CRTH2. Th cells from GD patients showed no significant changes between the two groups (Fig 3A–3D). In contrast, all of these markers were significantly higher on CD8 T cells (Fig 3E–3H). In addition, memory T cells were also investigated further for co-expression of inflammatory chemokine receptors CCR4, CCR5, CCR6 which are thought to define separate functional subsets Th2, Th1 and Th17 cells respectively [23, 24]. Increase in number of cells expressing CCR6 along with CCR4 and CCR5 was observed in GD compared to normal controls with a corresponding decrease in the fraction of CCR6- cells (Fig 4A–4E).

**Fig 5:**
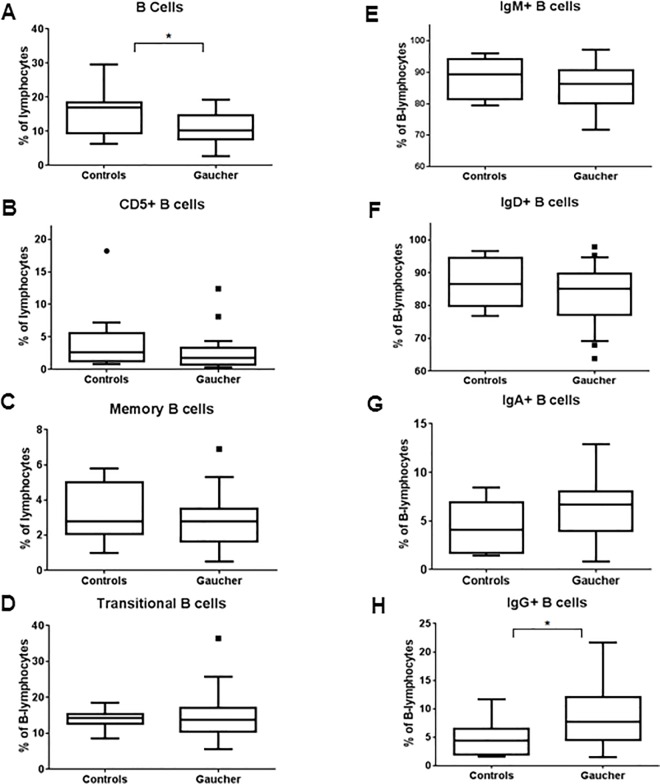
B-lymphocytes and subsets in GD patients. Percentage of B-lymphocytes (CD20+) from total lymphocytes of GD patients and normal controls were assessed using flow cytometry and plotted (A). B cells were further analyzed for presence of activation markers (CD5 and CD27). CD21 was used as B cell maturation marker where CD20+/CD21+ cells represented mature B cells while CD20+/CD21^Dim^ represent transitional B cells (A-D). B cells were subdivided into those producing individual immunoglobulins (IgA, IgG, IgM, and IgG) in GD patients and normal controls and plotted as % of B-lymphocytes (E-F).

B lymphocytes were identified by the expression of CD20 marker, while CD5 and CD27 were used as B cell activation and memory markers. While an overall decrease in B cells was observed in GD patients (P = 0.022), no significant changes were seen in activated and memory B cells (Fig 5A–5C). Immature, transitional and mature/marginal zone B cells were differentiated by the level of CD21 expression on CD20^+^ cells. Mature B cells show high level of CD21 expression (CD21^Hi^/CD20^+^) while immature B cells are enumerated as CD21^-^/CD20^+^ cells. There is a population of cells within peripheral blood with intermediate level of CD21 expression (CD21^Dim^/CD20^+^) which represents transitional B cells. When CD21^Dim^ cells were plotted for GD and controls, no significant change is observed when compared between the two groups. However, closer look at individual data points indicates a subgroup within GD patients which showed higher fraction of CD21^Dim^ cells indicating that in that subgroup of GD patients, defect in B cell maturation are observed (Fig 5D). B cells were then sub-divided into those producing each class of immunoglobulins, IgA, IgD, IgM and IgG. Initial observation noted a significantly higher IgG producing B cells in GD patients (P = 0.046), taking into consideration individual data points, there seem to be a trend for increased IgA producing cells and reduced IgD and IgM producing B cells (Fig 5E–5H).

**Fig 6:**
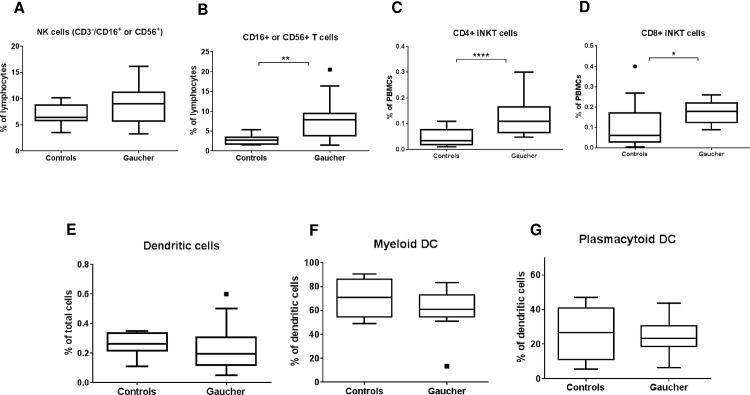
NK/NKT and dendritic cells in GD patients. NK cells (CD3-/ CD16+ or CD56+), NKT cells (CD3+/ CD16+ or CD56+) and invariant NKT cells (iNKT, CD3+, Vα24Jα18+, CD4+/CD8+) from peripheral blood of GD patients and normal controls were assessed using flow cytometry and plotted as percentage of total lymphocytes (A-D). Dendritic cells were enumerated as Lin-/CD34-/HLA DR+ cells and plotted as a percentage of total leukocytes/WBCs (E). Dendritic cells were further fractionated as myeloid DCs or plasmacytoid DCs based on expression of CD11c and BDCA2 respectively and plotted as percentage of DCs (F&G).

Natural killer cells (NK cells) are identified as CD45+ lymphocytes that express CD16 or CD56 and lack CD3 expression. No significant change in NK cell fraction was seen between GD and control groups, even though there seems to be a trend of increased NK cells in GD (Fig 6A). CD3 expressing NK cell fraction was markedly higher in GD group compared to non GD controls (P = 0.0053) (Fig 6B). The invariant NKT cells (iNKT cells) are a subgroup of NKT cells which express an invariant T cell receptor α-chain, Vα24-Jα18. Subgroups of iNKT cells expressing CD4 and CD8 are both significantly increased in the GD group (Fig 6C&6D). Dendritic cells were identified as those leukocytes which are lineage-1 negative (CD3, CD14, CD16, CD19, CD20 and CD56 negative), CD34 negative and HLA DR positive. DCs were further classified as myeloid and plasmacytoid DCs by their expression of CD11c or BDCA2 respectively. No significant change in DC fraction was observed between GD and control group however in a subgroup of GD patients there were no or very low DCs (Fig 6E) There were no overall significant changes in myeloid and plasmacytoid DC numbers as well (Fig 6F&6G).

### Time of initiation of therapy (ERT) and disease severity correlate with persistent immune alterations in GD

**Fig 7:**
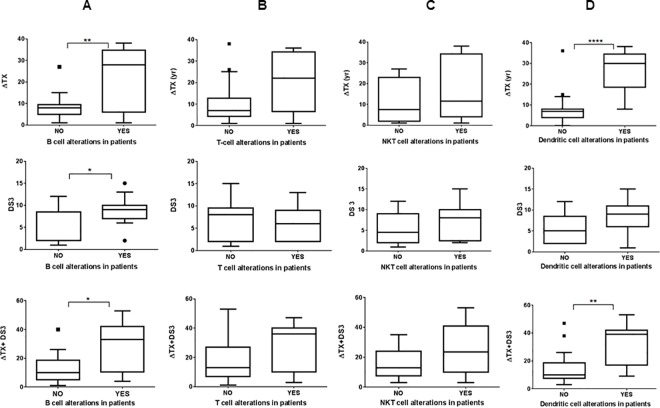
Correlation between immune alterations and ΔTX, DS3 and combined scores. GD patients were sub-categorized based on whether or not they show persistent B-cell (A), T-cell (B), NKT cells (CD16/CD56+ T cells) (C) and dendritic cell (D) immune alterations and plotted against the delay in initiation of ERT (ΔTX), disease severity (DS3) and a combined score (ΔTX+DS3).

GD patients were subdivided into two groups based on whether they showed persistent immune alterations which include one or more of the following B-lymphocyte abnormalities: overall decrease in B cell fraction, increased IgA or IgG producing subclasses, B cell maturation defects as seen by abnormal increase in number of B cells expressing low CD21 marker and corresponding decrease in CD21+ B cells. Individual ΔTX, DS3 scores as well as combined scores were plotted for the two groups: patients manifesting B cell abnormalities or those who were not. ΔTX of the group with B cell immune alterations was significantly higher (P = 0.0028) compared to those lacking B cell abnormalities indicating that delay in the initiation of therapy has profound effect on persistent B cell alterations in GD patients. Similar effect was found with DS3 scores (P = 0.01) as well as combined scores (P = 0.01) for each group (Fig 7A). When similar analysis was performed by subdividing the GD patients based on their overall T cell abnormalities, which included skewed T4/T8 ratio, abnormally high CD8 fraction, or increased number of activated T cells, no significant effect of either ΔTX or DS3 was observed (Fig 7B). There was no significant difference between the patients with or without the NKT cell alterations in patients with different ΔTX and DS3 (Fig 7C). These results suggest that T cell and NKT cell abnormalities in GD patients are not profoundly affected by the delay in treatment initiation. GD patients were further sub-grouped based on presence or absence of normal range of dendritic cells and analyzed for their ΔTX. There is a highly significant correlation (P<0.0001) between the reduced number of DCs and delay in treatment initiation. Similar effect was seen with combined score (P = 0.0034) (Fig 7D).

### Delay in treatment initiation affects various immune cell subsets in GD patients

**Fig 8:**
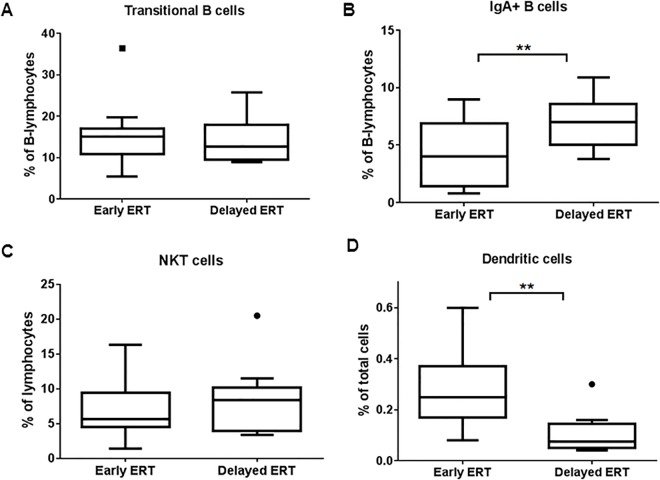
Effect of early or delayed therapy on immune irregularities. GD patients were sub-divided into those who received early vs. delayed intervention after their diagnosis and plotted for specific immune phenotypes. B cell transitional cells expressed as CD21^Dim^ (A), IgA producing B cells (B), NKT cells (C) and dendritic cell (D) percentages in GD patients with delayed or early intervention are plotted.

GD patients were sub-divided into those who received early (<7 yr.) vs. delayed (>8 yr.) intervention after the onset of symptoms and plotted for affected immune phenotypes as seen above. Transitional B cells as seen by CD20/ CD21^**Dim**^ expression were higher in patients with delayed ERT compared to those with early intervention. IgA-producing B cells were also reduced as a result of early ERT (P = 0.0046) (Fig 8A and 8B). The change in NKT cell fraction was less prominent between the groups with early and delayed ERT while the effect on DCs percentages was much more pronounced (P = 0.0017) (Fig 8C and 8D). Taken together these results indicate that initiating the therapy early in GD patients helps to revert some of the long term immune cell abnormalities.

## Discussion

Most of the studies exploring immune irregularities in GD have been focused on dysregulation of macrophages due to the fact that the accumulation of sphingolipids is most prominent in cells of monocyte/macrophage lineage [1]. Prior B cell associated studies in GD patients have been focused on B cell malignancies and myelomas [4, 25]. However it is important to investigate how various cell types in innate and adaptive immune system are affected to better understand the cellular pathophysiology of GD. A systematic clinical study involving detailed immunophenotyping of individual GD patients revealed that long term and persistent alterations exist in all major immune components albeit significant effects are seen in certain subsets. While there were abnormalities observed in overall number of B and T-lymphocytes, NKT cells and dendritic cells are affected as well. In addition, significant differences were also seen in activated T cells, memory markers as well as B cell maturation and development of memory B cells are affected as well. Overall increase in CD20^+^/CD27^+^ fraction indicates impaired development of memory B cells in GD patients. IgM producing B cells were in similar range compared to non GD controls, however there were marked alterations in IgA and IgG producing B-cells. However, pneumococcal titers for a majority of patients were found to be in the normal range (data not shown) thus ruling out antibody deficiencies *per se*. CD3+ T cells were assayed for various additional markers revealing interesting differences between GD and control groups. There was a reduction in CD4+ T helper cells with a corresponding increase in CD8+ cytotoxic T cells thus skewing the CD4/CD8 ratio which is generally considered as an indicative of immune health. Assays that include T cell activation markers revealed an overall increase in activated T cells as well as increase expression of chemokine markers in GD patient group. While B-cell malignancies are definitely a concern in GD patients; our study highlights the fact that irrespective of clinical manifestations, there are multiple persistent abnormalities pertaining to immune system in GD patients.

In order to better understand the basis of persistent multiple immune irregularities, we subdivided the GD group into those manifesting significant immune alterations and those which do not carry any, and analyzed the time of disease onset and delay in therapy initiation (ΔTX) in each subgroup. In case of T cell abnormalities, even though there is a trend, there is no statistically significant correlation with ΔTX. However, in all other cell types including B-lymphocytes and DCs, a significant correlation was observed with ΔTX. We then found similar results using DS3 and combined score values as well indicating that having immune alterations contributes to overall disease manifestation and severity in spite of continuing ERT. While dendritic cells primarily act as antigen presenting cells, they have been shown to play important roles in activating T-lymphocytes [26, 27]. Reduced number of circulating DCs has been indicated in various disease states including immune disorders [28]. We observed a strong correlation between reduced DCs in patients with severe GD pathology even though the exact mechanism it yet to be elucidated. Alternately the GD group was divided into those who received early ERT compared to delayed ERT and looked for persistence of various immune alterations in each group. As expected, early initiation of therapy was found to reverse most of the immune alterations. Our study highlights the severity of immune abnormalities and its correlation with manifestation of symptoms in GD patients and advocates initiating treatment early in order to reverse these irregularities and avoid persistent effects.

A small cohort size and broad definitions, such as the treatment lag (ΔTX) in clinical assessments are some of the limitations. However, this study data demonstrates that there are quantitative variations in immunologic cell types existing in patients with GD who are receiving ERT. Patients who were diagnosed before and started therapy after the advent of ERT, thus already had significant disease load and suffering from complications, especially of skeletal in origin. Patients diagnosed after the advent of ERT were able to start receiving ERT before the severe complications ensued. Given that there are limitations of ERT for access to different tissues and organs, the outcome measures currently in use have similar limited expectations, whilst only less than half of the treated patients achieve all established treatment goals. Compared to earlier, currently there are more individuals being identified with GBA mutations at asymptomatic or minimally symptomatic stages, and more patients with GD who were able to start therapy soon after their diagnosis in childhood. Finally, while GD is not a primary immunologic disorder, the involvement and permanent changes in multiple immune cell lineages expand the effects of the sphingolipid metabolism and/or GBA mutations beyond the macrophage lineage.

## Supporting Information

S1 Fig Flow cytometry analysis to identify lymphocyte subpopulations. Peripheral blood cells expressing high CD45 were gated as lymphocytes. T- and B-lymphocyte fractions were enumerated based on the expression of CD3 and CD20 markers respectively. Similarly, NK cells were identified as those expressing either CD16 or CD56 while being negative for CD3 expression.
(TIF)

S2 Fig Subpopulations within T-lymphocytes. T cells were gated based on CD3 expression and further classified into cytotoxic T cells (Tc) and T helper cells (Th) based on the markers CD8 and CD4 respectively. Memory CD8 and CD4 T cells were identified based on CD45RO expression. Expression of CD194 and CD183 in each of the T cell fractions is noted.
(TIF)

S3 Fig Subpopulations within B-lymphocytes. Activated B cells were identified as CD20+ cells expressing activation markers CD5 or CD20. Level of CD21 coexpression on CD20+ cells was used to mark B cell stages. CD20/CD21^Hi^ cells are mature B cells while CD20/CD21^dim^ cells indicate transitional B cells.
(TIF)

S4 Fig B cell fractions producing various immunoglobulin (Ig) subtypes. B cells were gated based on either CD20 or CD19 expression, which are coexpressed on almost B cells. Mature B cells producing individual immunoglobulin subtypes are identified by surface expression of those Igs.
(TIF)

S5 Fig Dendritic cells and subpopulations. Dendritic cells were identified as cells with are negative for lineage cocktail (CD3, CD14. CD16, CD19, CD20, CD56) and CD34 while expressing HLA-DR. DCs are further classified into myeloid and plamsacytoid DCs based on CD11c and BDCA expression respectively.
(TIF)

S1 Table Immunophenotyping results in GD subjects and controls. Immune subsets from immunophenotyping using flow cytrometry are presented. All the results, except CD4/CD8 ratio are expressed as percentages. T-, B- and NK cells are all expressed as fraction of peripheral blood lymphocytes (CD45+). Dendritic cells are shown as percentage of total leukocytes.
(TIF)

## Abbreviations

GD: Gaucher disease
NK cells: Natural killer cells
APCs: Antigen presenting cells
ERT: Enzyme replacement therapy
DS3: Disease severity score
ΔTX: Delay in initiation of therapy
ICF: Informed consent form
PBMCs: Peripheral blood mononuclear cells
M: Male
F: Female
SX: Age at which symptoms manifested
TX: Age at which treatment for GD was initiated
Th: T helper cells
Tc: Cytotoxic T cells
Ig: Immunoglobulin
NKT cells: Natural killer T cells
iNKT cells: invariant Natural killer T cells
DCs: Dendritic cells
WBCs: White blood cells
IRB: Internal review board
