# Supplementary figures and images for: Time of Initiating Enzyme Replacement Therapy Affects Immune Abnormalities and Disease Severity in Patients with Gaucher Disease
Source: PLoS One. 2016 Dec 12;11(12):e0168135. doi: 10.1371/journal.pone.0168135 (PMC5152900; doi:10.1371/journal.pone.0168135)

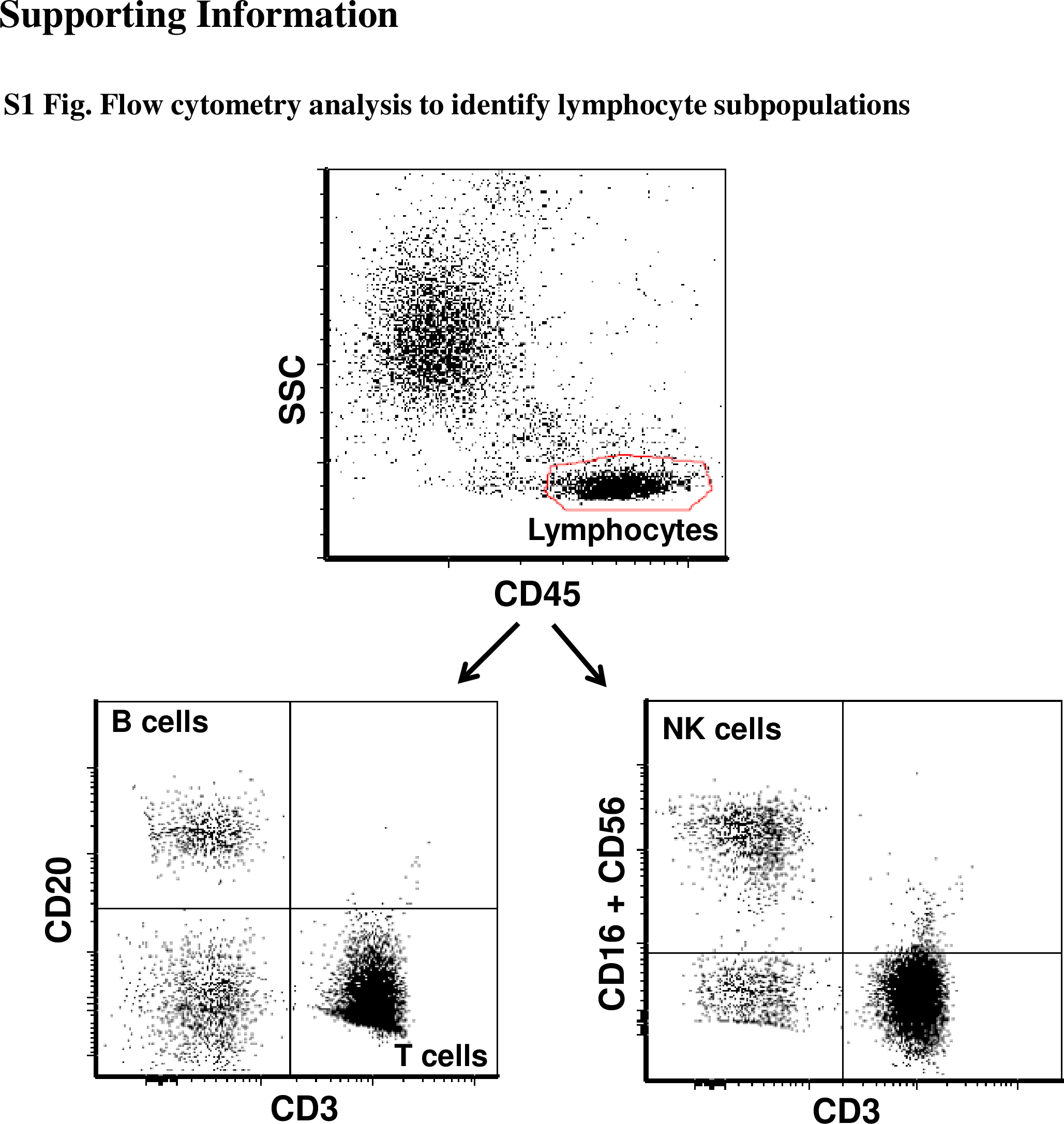

Supplement: S1 Fig — Peripheral blood cells expressing high CD45 were gated as lymphocytes. T- and B-lymphocyte fractions were enumerated based on the expression of CD3 and CD20 markers respectively. Similarly, NK cells were identified as those expressing either CD16 or CD56 while being negative for CD3 expression. (TIF) [file pone.0168135.s001.tif]

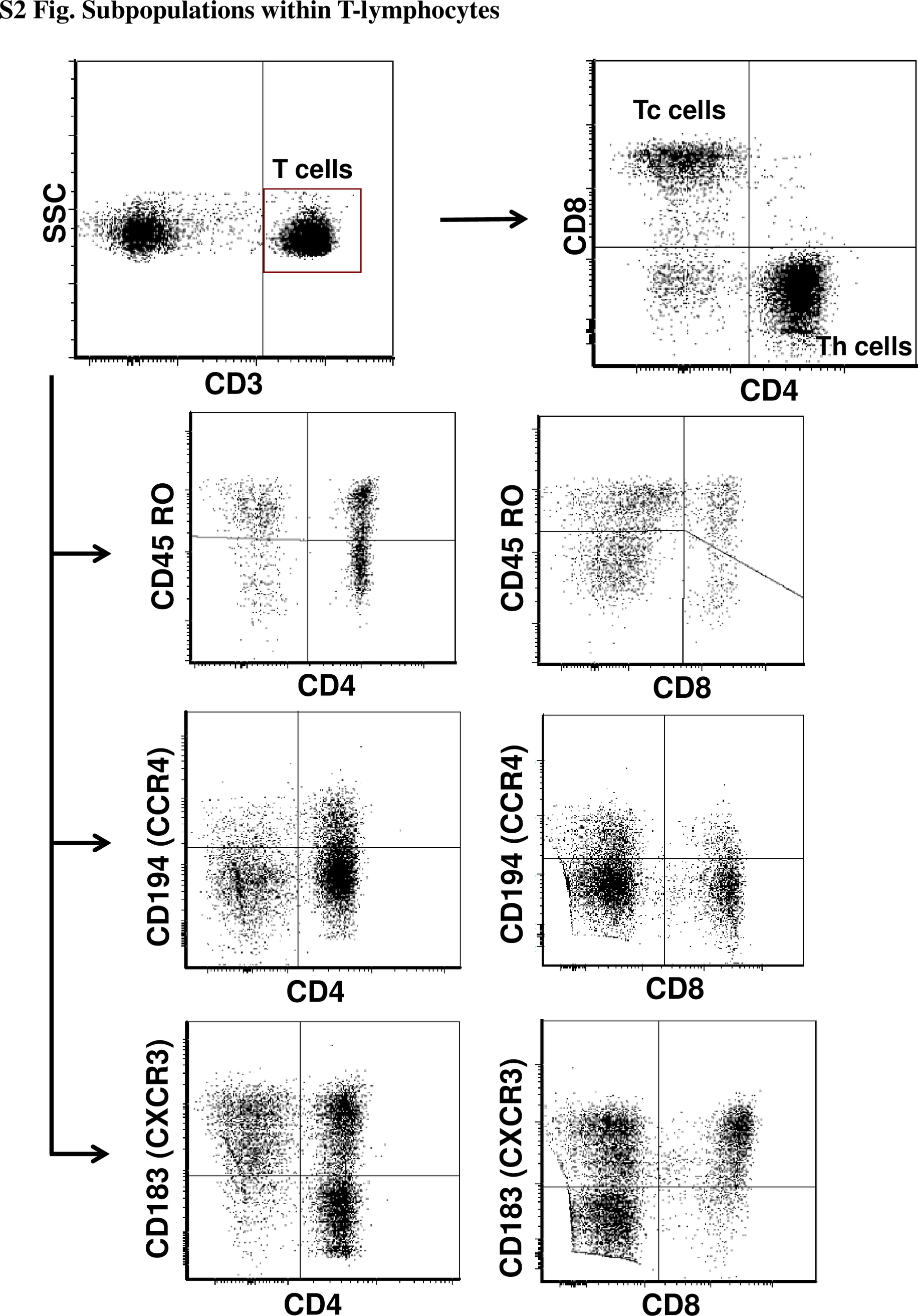

Supplement: S2 Fig — T cells were gated based on CD3 expression and further classified into cytotoxic T cells (Tc) and T helper cells (Th) based on the markers CD8 and CD4 respectively. Memory CD8 and CD4 T cells were identified based on CD45RO expression. Expression of CD194 and CD183 in each of the T cell fractions is noted. (TIF) [file pone.0168135.s002.tif]

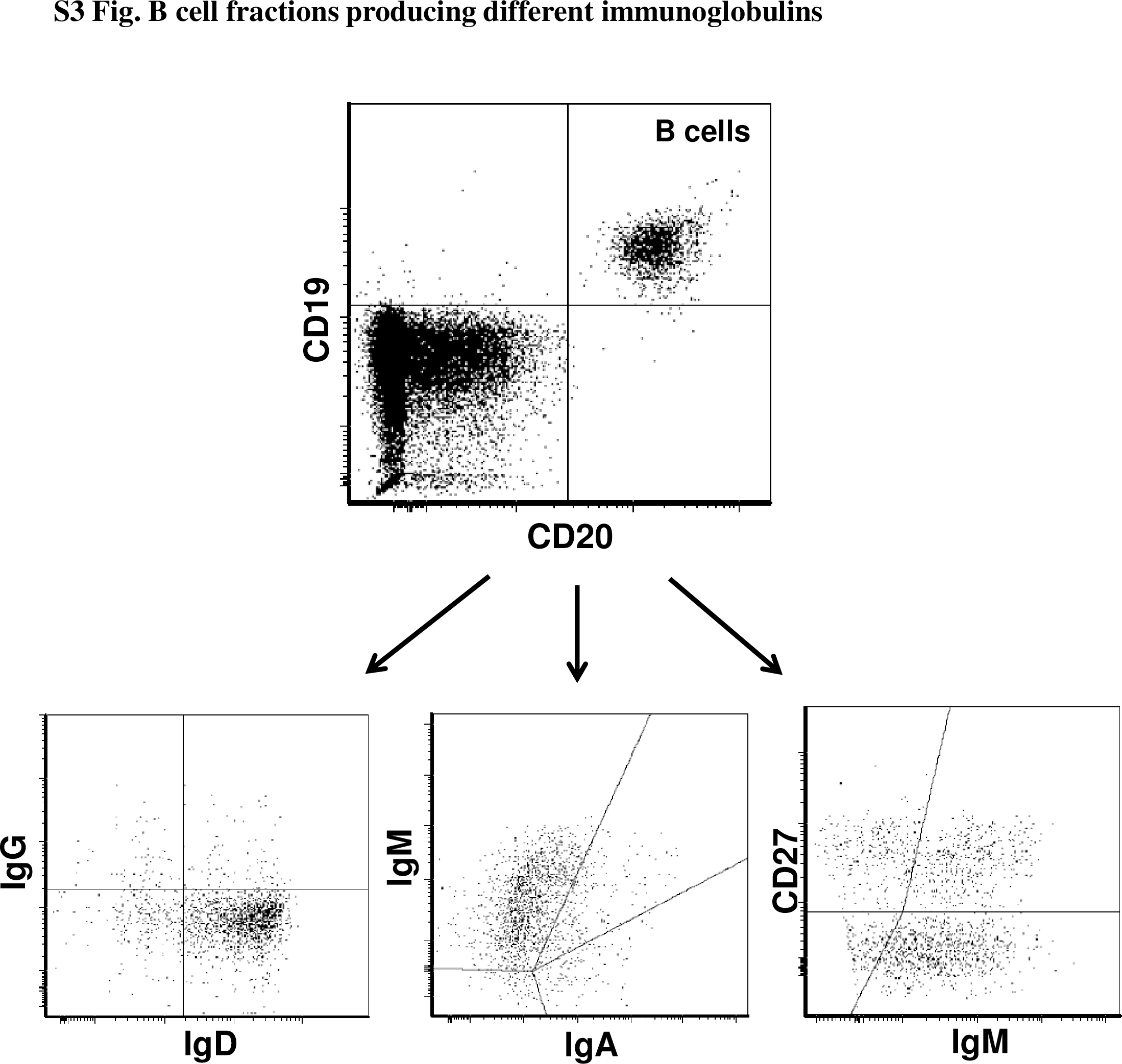

Supplement: S3 Fig — Activated B cells were identified as CD20+ cells expressing activation markers CD5 or CD20. Level of CD21 coexpression on CD20+ cells was used to mark B cell stages. CD20/CD21Hi cells are mature B cells while CD20/CD21dim cells indicate transitional B cells. (TIF) [file pone.0168135.s003.tif]

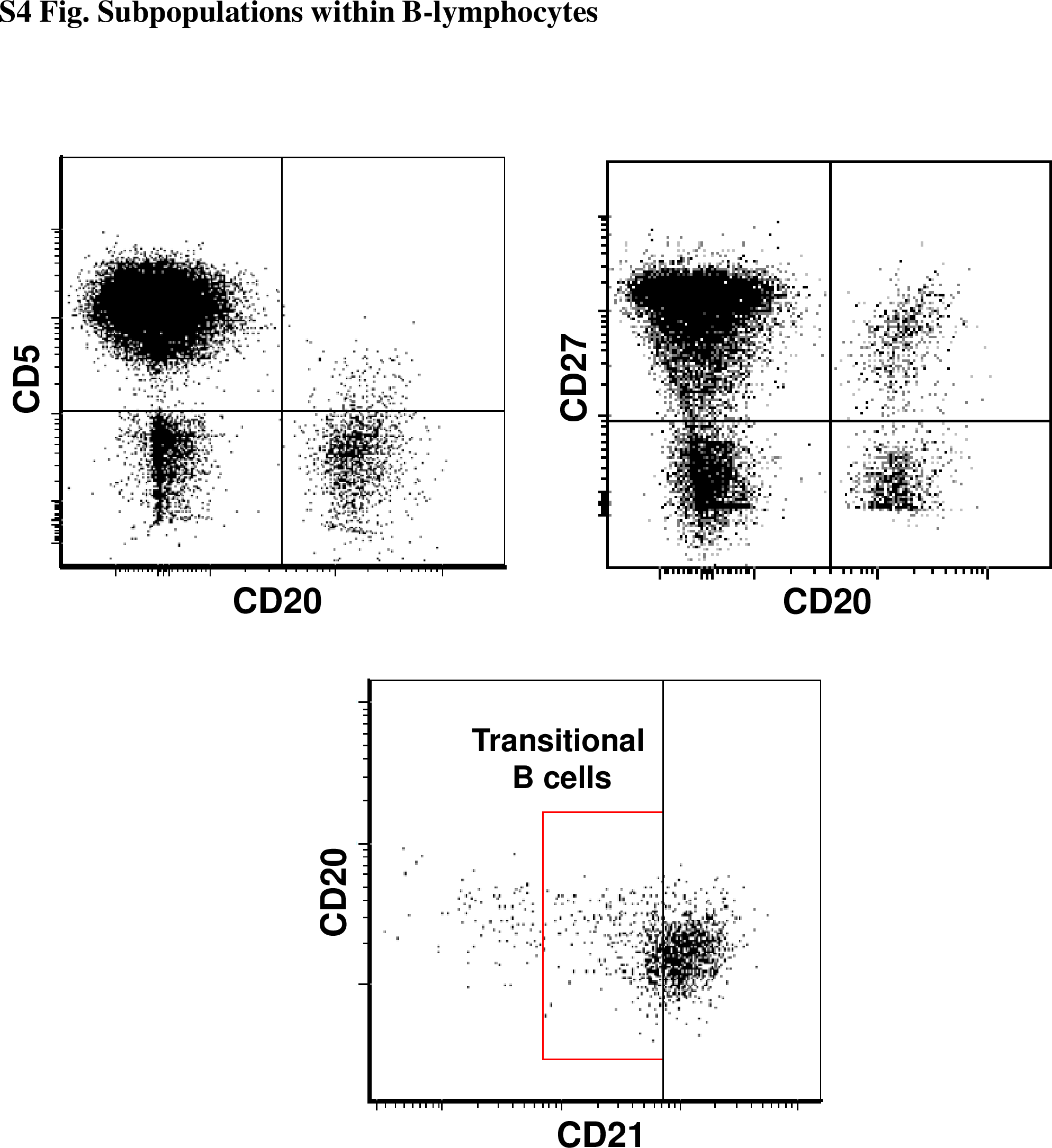

Supplement: S4 Fig — B cells were gated based on either CD20 or CD19 expression, which are coexpressed on almost B cells. Mature B cells producing individual immunoglobulin subtypes are identified by surface expression of those Igs. (TIF) [file pone.0168135.s004.tif]

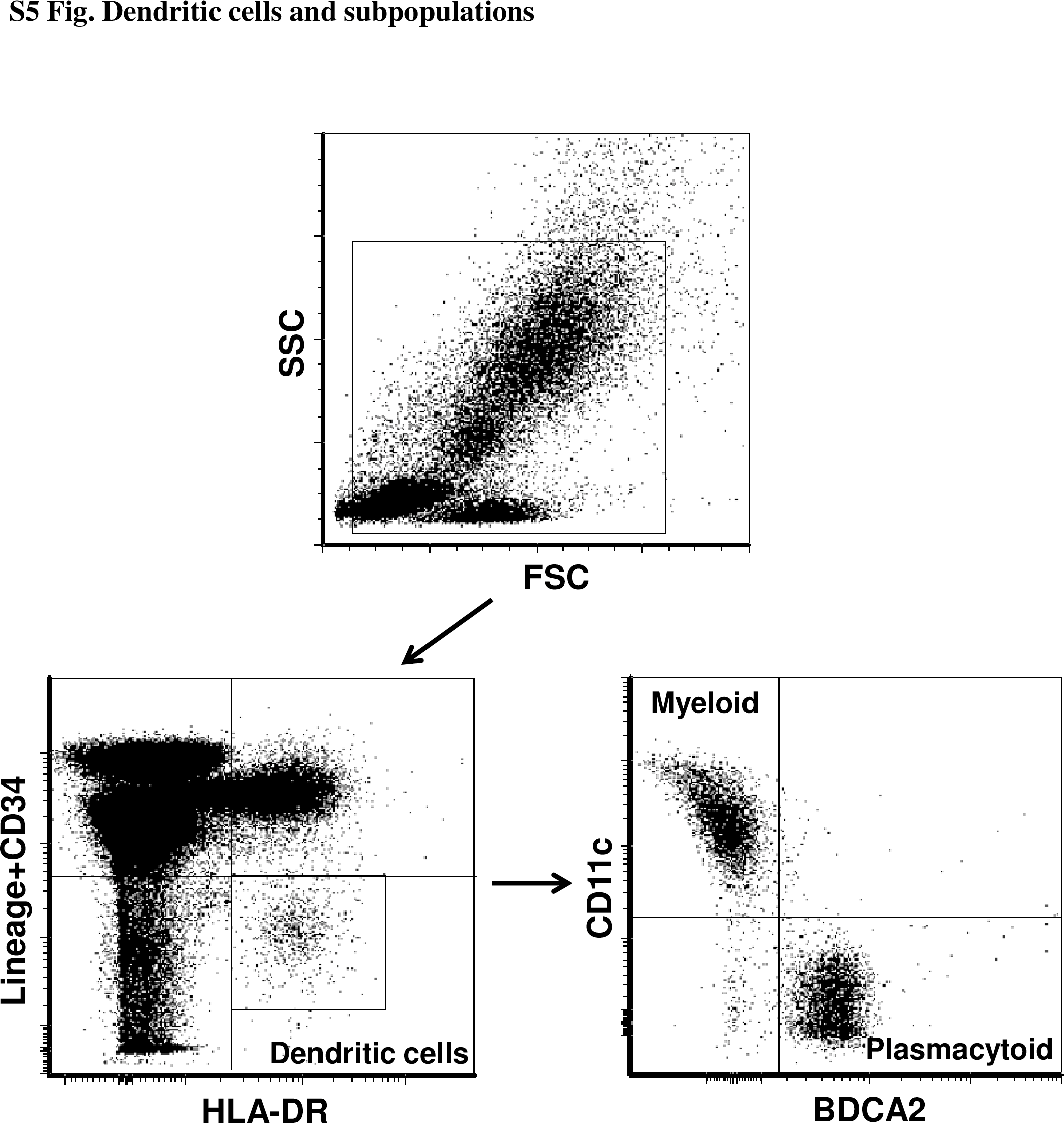

Supplement: S5 Fig — Dendritic cells were identified as cells with are negative for lineage cocktail (CD3, CD14. CD16, CD19, CD20, CD56) and CD34 while expressing HLA-DR. DCs are further classified into myeloid and plamsacytoid DCs based on CD11c and BDCA expression respectively. (TIF) [file pone.0168135.s005.tif]

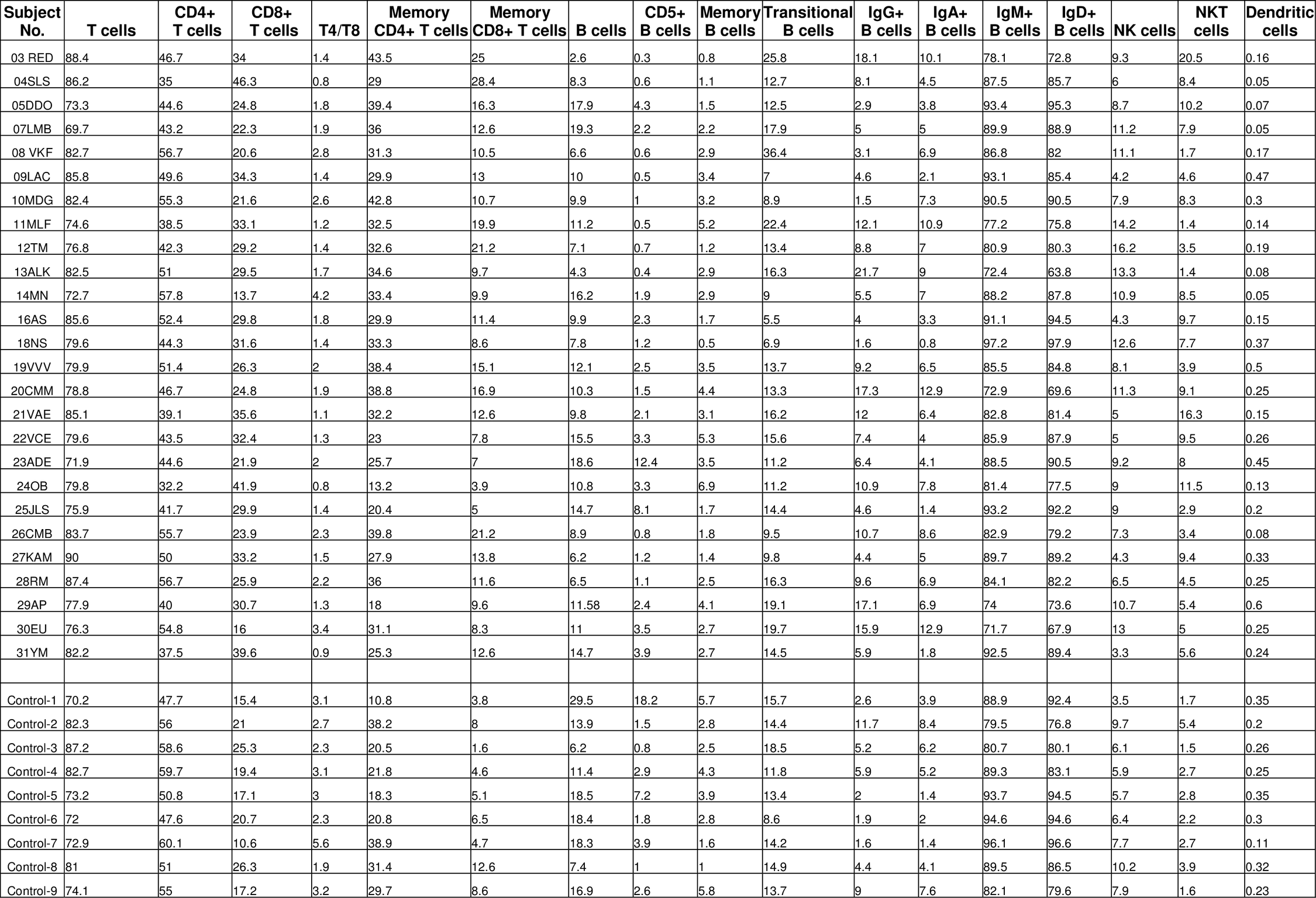

Supplement: S1 Table — Immune subsets from immunophenotyping using flow cytrometry are presented. All the results, except CD4/CD8 ratio are expressed as percentages. T-, B- and NK cells are all expressed as fraction of peripheral blood lymphocytes (CD45+). Dendritic cells are shown as percentage of total leukocytes. (TIF) [file pone.0168135.s006.tif]
